# Perioperative Opioid Exposure Patterns in Pediatric Anterior Cruciate Ligament Reconstruction: A Ten-Year Administrative Database Study

**DOI:** 10.7759/cureus.13927

**Published:** 2021-03-16

**Authors:** Dharman Anandarajan, Brendan A Williams, Nathan D Markiewitz, Divya Talwar, Lawrence Wells

**Affiliations:** 1 Division of Orthopaedics, Children's Hospital of Philadelphia, Philadelphia, USA

**Keywords:** acl, opioids, hospital, adolescents, pediatrics

## Abstract

Introduction: Variation in opioid exposure has been documented in many pediatric fields; however, little is currently known about the extent of these findings during the perioperative period. The purpose of this study was to examine perioperative opioid exposure on a national level among patients undergoing anterior cruciate ligament (ACL) reconstruction using an administrative database. Our aims were to assess the impact of hospitals and a variety of demographic factors on (1) the likelihood of perioperative opioid exposure and (2) the variability in relative opioid exposure.

Methods: The Pediatric Health Information Systems Database (PHIS) was used to identify pediatric patients (≤ 18 years old) across 52 hospitals undergoing ACL reconstruction between January 2008 and December 2017. Administered opioids in morphine milligram equivalents were discretized into quintiles to represent relative opioid exposure (ROE). A hurdle generalized additive model was estimated to identify demographic factors predictive of (1) the receipt of any opioid medication and (2) the ROE among those receiving opioids.

Results: Of the 19,821 patients meeting study inclusion criteria, 17,350 (88%) were administered opioid medications perioperatively. There was no temporal trend in perioperative opioid utilization or ROE over the study period. Patients in an inpatient (OR = 0.260 [0.221, 0.305]) or observation unit (OR = 0.349 [0.305, 0.401]) context were less likely to be administered opioids. Female patients (OR = 0.896 [0.813, 0.987]) were less likely to be administered opioids, while patients on commercial insurance had a higher ROE (OR = 1.09 [1.023, 1.161]). Patient age and hospital-level time trends predicted opioid administration and exposure (max p < 0.001).

Discussion: Gender, age, surgical setting, hospital type, and insurance status, in part, predicted perioperative opioid exposure among pediatric patients undergoing ACL reconstruction surgery. Exposure has not declined in recent years and varies significantly between hospitals. Although this study primarily served to document demographic variability in perioperative opioid exposure in pediatric patients undergoing ACL reconstruction, the understanding of variability in perioperative opioid utilization and exposure rate could stand to be further explored.

## Introduction

Patients requiring orthopedic intervention are subject to opioid exposure as pain management in the setting of acute injury and post-operative period remain particularly opioid-centric [1,2]. While recent studies have focused on demographic factors impacting pediatric opioid exposure in orthopedics and other pediatric surgical fields [3-6], little study of perioperative orthopedic opioid exposure exists to the best of our knowledge. Increased understanding of nationwide opioid utilization patterns during these early phases of care is essential to grasping the entire scope of the pediatric opioid utilization and exposure in our country.

Anterior cruciate ligament (ACL) tears are one of the most common sports-related injuries in the pediatric population and are occurring with increased frequency due, in part, to increased sports specialization and year-round participation [7]. With the hopes of preventing the long-term sequelae of an ACL-deficient knee, surgical intervention for ACL injuries has also increased in recent years [8]. Consequently, rigorous evaluation of perioperative opioid administration and potential nationwide variability in exposure is of utmost importance to increase the physician awareness of their patient’s cumulative opioid exposure.

The purpose of this study was to detail pediatric perioperative opioid utilization patterns among patients undergoing ACL reconstruction on a national level. We sought to examine temporal trends in perioperative opioid utilization, determine if patient characteristics are predictive of perioperative opioid utilization, and if variability in opioid utilization persists after adjustment for those characteristics. Given the documented variation of opioid administration and utilization in other pediatric fields [4,9], we hypothesized that (1) patient characteristics will predict the likelihood and relative perioperative opioid utilization; (2) perioperative opioid utilization rates will vary between hospitals even after adjustment for patient characteristics; and (3) perioperative opioid utilization has decreased in recent years.

## Materials and methods

Data source

This study utilized the Pediatric Health Information System Database (PHIS), a comprehensive clinical and financial information database. Developed by the Children’s Hospital Association (Lenexa, KS), data within PHIS are derived from not-for-profit, freestanding, tertiary care pediatric centers. PHIS can be queried for data regarding patient encounters such as clinical diagnosis, procedures, and patient demographics. In addition, the database houses resource utilization parameters such as imaging, pharmacy, clinical, and supplies.

Population and selection criteria

The patient cohort for this study consisted of pediatric patients ≤ 18 years old operatively treated for a diagnosis of an ACL tear between January 2008 and December 2017. Patients were identified by International Classification of Diseases (ICD)-9, ICD-10, ICD Procedure Coding System (PCS)-9, ICD PCS-10, and Current Procedural Terminology (CPT) codes following methodology reported in a prior PHIS study on ACL injury by Tepolt et al. [8] Those with a diagnosis of a discoid lateral meniscus tear, a congenital absence of the ACL, a congenitally short femur, a posterior cruciate ligament (PCL) tear, or a tibial spine avulsion were excluded from this study. Patient characteristics included age, year of procedure, race (Asian vs. Black vs. Multiracial vs. Others vs. White), primary payer (commercial vs. noncommercial primary payer), and surgical setting (inpatient vs. observation unit vs. ambulatory).

Measures of opioid exposure

Perioperative opioid exposure was examined, defined as all opioid-class medication administered to the patient while at the hospital or ambulatory surgery center from the day of surgery until the time of discharge. Although the Medication Administration Record (MAR) is not captured by PHIS, prior studies have demonstrated a high concordance between pharmacy administrative billing data and the MAR for other therapeutic agents [10,11]. Thus, we used PHIS medication billing as a proxy for actual administration of medication to the patient. Outpatient opioid prescription data is not available in the PHIS database and was not the focus of this study.

Each patient encounter was reviewed for all in-hospital medications including type, dosage, and estimated duration. Given the wide variety of opioid medications and doses administered, we began by converting all opioids reported during each patient encounter to an oral morphine equivalent dose, thereby creating a standardized estimate of total potential opioid exposure. The contribution of each medication was calculated over the perioperative period by multiplying the dose of each opioid by its duration and a conversion factor and then summing the consequent values for each patient [12-14]. Patients administered opioids with unreported doses or administration routes were removed from the analysis (n = 246 [1%]).

Given the limitations of the database’s medication duration estimate (PHIS rounds up to full-day durations) and the lack of distinction between administered and actual delivered dose, this calculation is certainly an overestimate of opioid exposure. Even with exact dosing information, the calculation of an oral morphine equivalent is often considered an oversimplification. The uncorrected use of oral morphine equivalents in this setting would overstate the reliability and validity of our measurement. To that end, we grouped opioid-exposed patients by oral morphine equivalents into quintiles, creating the ordinal variable relative opioid exposure (ROE). We believe the use of ROE balances robustness and sensitivity in making relative comparisons in total exposure between patients managed with different pharmacologic approaches across hospitals and time.

Statistical analysis

All analyses were performed in R version 3.5.3 using mgcv. Descriptive statistics of the demographic variables and perioperative opioid dosing of our sample are reported. A hurdle generalized additive model (GAM) was then used to model the relationship between demographic variables and opioid exposure. A hurdle model describes the process that produces non-zero values (i.e., the decision to administer opioids) and separates from the process that determines the magnitude of that non-zero value (i.e., the decision to administer that number of opioids, conditional on opioid administration). GAMs allow for the penalized estimation of smooth functional relationships (“smooths”) between continuous variables and the outcome of interest (e.g., age and ROE). We included parametric fixed effects of race, primary payer, and surgical setting, as well as smooth fixed effects of age and year of operation. Random smooth trajectories for each hospital were also modeled to account for unmeasured differences in hospital-level administration practices. We estimated the binary process through a binomial distribution with a logit link function and the ordinal process through an ordered categorical model logistic link.

## Results

**Figure 1:**
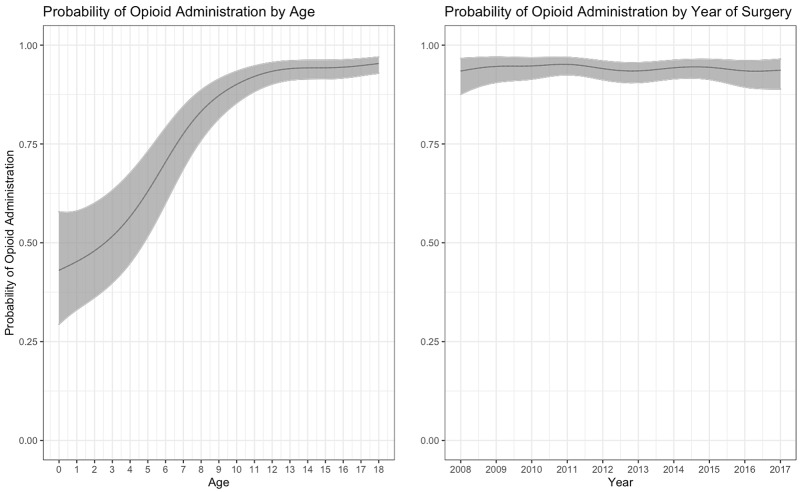
Probability of Opioid Administration by Age and Year With 95% Confidence Intervals

**Figure 2:**
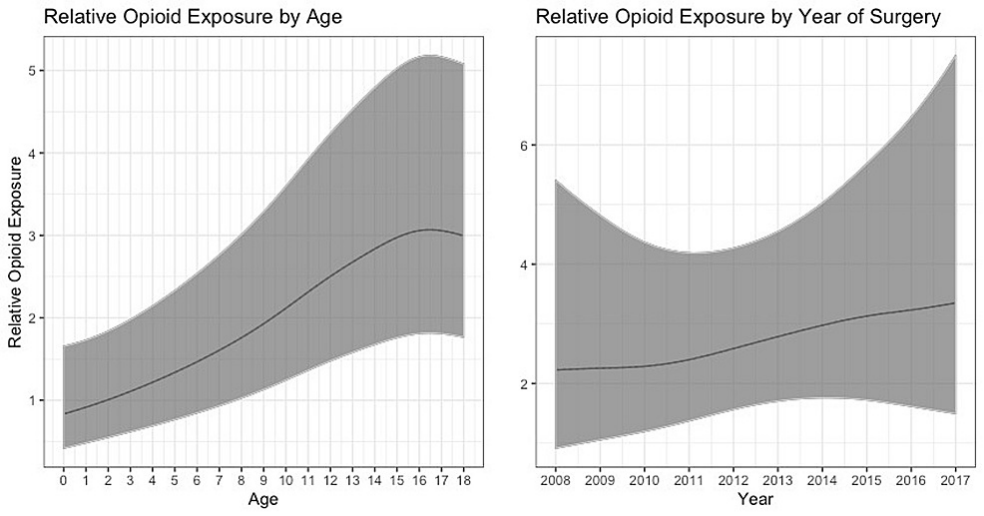
Relative Opioid Exposure by Age and Year With 95% Confidence Intervals

**Figure 3:**
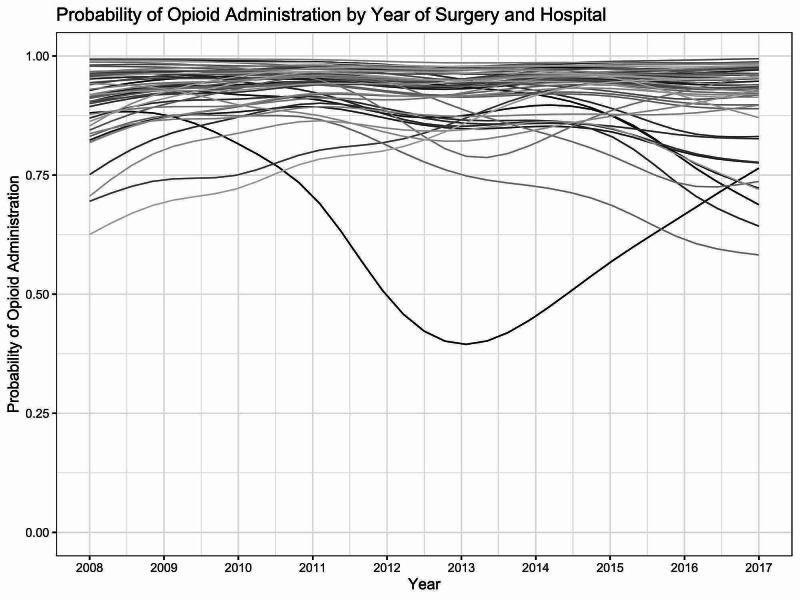
Hospital Trajectories of Probability of Opioid Administration

**Figure 4:**
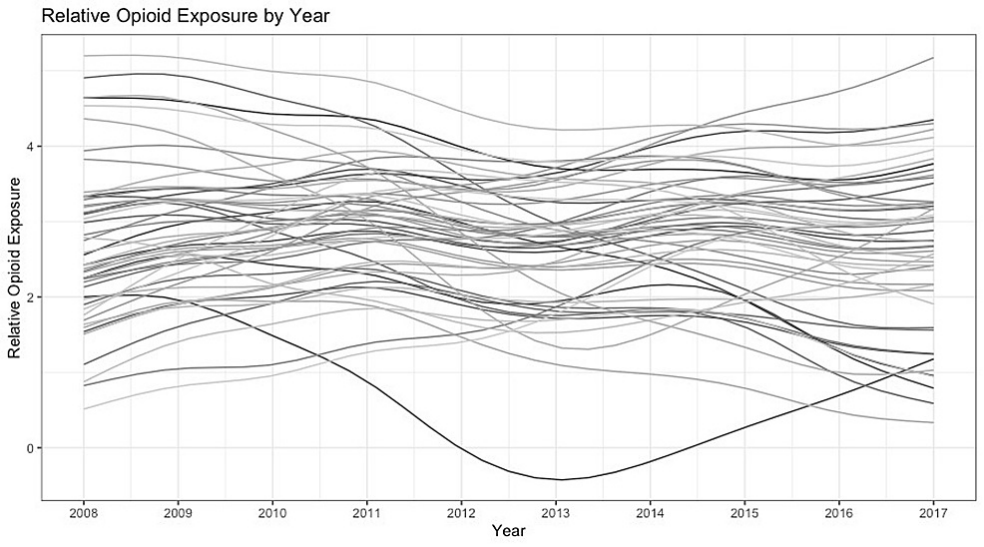
Hospital Trajectories of Relative Opioid Exposure

After appropriate exclusions, we identified 19,821 patients meeting criteria over the defined study period. Perioperative opioid exposure was identified in 17,350 (88%) patients. Descriptive statistics are detailed in Table 1. All models converged, and diagnostics demonstrated that enough flexibility was provided to capture the smooth relationships. We present parametric model results in Table 2 and smooth relationships in Figures 1-4.

**Table 1:** Summary Statistics for Predictors and Outcomes All variables are summarized as number and percent except ^a^mean and standard deviation.

Variable		Summary Statistics
Age^a^		14.2 (3.1)
Opioid exposure		
	Perioperative opioids	17,350 (88)
	No perioperative opioids	2,471 (12)
Race		
	Asian	345 (2)
	Black	3,335 (17)
	Other	4,203 (21)
	Multiracial	90 (<1)
	White	11,848 (60)
Gender		
	Male	10,611 (54)
	Female	9,210 (46)
Setting		
	Ambulatory	15,379 (77)
	Inpatient	1939 (10)
	Observation	2,503 (13)
Payer		
	Noncommercial	9,462 (48)
	Commercial	10,359 (52)

**Table 2:** Odds Ratios of Parametric Predictors in the Hurdle GAM Predicting Morphine Equivalent Dose in Pediatric Patients Undergoing ACL Reconstruction Model variables with 95% confidence intervals not including 1 are indicated with an * and bolded to indicate significance. GAM, Generalized additive model; ACL, anterior cruciate ligament.

		Model 1: Logistic Regression	Model 2: Ordinal Regression
Race			
	White	Reference	
	Asian	0.786 [0.556, 1.111]	0.845 [0.681, 1.050]
	Black	1.048 [0.907, 1.211]	1.059 [0.977, 1.148]
	Multiracial	0.587 [0.307, 1.121]	1.205 [0.781, 1.860]
	Other	1.028 [0.892, 1.184]	1.012 [0.932, 1.098]
Gender			
	Male	Reference	
	Female	0.896 [0.813, 0.987]*	1.018 [0.962, 1.076]
Surgical setting			
	Ambulatory	Reference	
	Inpatient	0.260 [0.221, 0.305]*	0.987 [0.862, 1.130]
	Observation unit	0.349 [0.305, 0.401]*	1.001 [0.896, 1.119]
Primary payer			
	Noncommercial		
	Commercial	1.094 [0.979, 1.224]	1.009 [1.023, 1.161]*

Predicting the administration of opioids

The first part of the hurdle model predicted which patients were administered opioids. Female patients (OR = 0.896 [0.813, 0.987]) and patients who were treated in an inpatient unit (OR = 0.260 [0.221, 0.305]) or observation unit (OR = 0.349 [0.305, 0.401]) setting were less likely to be administered opioids. We also found significant smooth relationships of patient age (p < 0.001) and random hospital-level trends (p < 0.001). The smooth relationship of year of procedure and opioid administration was insignificant (p = 0.114).

Predicting relative opioid exposure

The second part of the hurdle model predicts the ROE of patients who were administered opioids. Patients with commercial insurance (OR = 1.09 [1.023, 1.161]) had a higher ROE. The effect of race, gender, and an observation unit setting were all insignificant (min p = 0.13). The smooth relationships of both patient age and hospital-level trends with ROE were significant (max p < 0.001), an indication that both of these factors played a role in likelihood of opioid utilization. The smooth relationship between year of procedure and ROE was insignificant (p = 0.74), an indication that perioperative opioid utilization did not decrease over the studied timeframe.

## Discussion

This is one of the rare studies to examine the perioperative opioid exposure in pediatric patients undergoing ACL reconstruction surgery using a nationwide database. We found significant differences in the likelihood of opioid exposure based on gender and surgical setting. We estimated a significant difference in ROE based on primary payer. Perioperative opioid utilization as measured by both of our exposure variables (oral morphine equivalent and relative morphine exposure) did not appear to change for the typical hospital over the study period. We report our findings as differences due to setting, patient characteristics, and hospital-specific trends. Although it is important to keep in perspective that perioperative opioid utilization is largely dependent on an individual patient’s needs and will always vary on a case by case basis, our findings indicate that certain demographic factors may still have a role in determining perioperative opioid utilization.

Study results indicated that female patients were less likely to be administered opioids but were administered them at comparable ROE to male patients. Reasons for our findings regarding gender are less clear. While the mechanism of injury and timing of surgery may prompt different administration of these medications, implicit bias could also explain the reduced rate of opioid exposure for female patients. Epidemiological studies of gender disparities in the treatment of pain paint a mixed picture [15-17]. As we found a gender difference in opioid exposure, but not dose, our results are suggestive of potential gender disparity in pain management during ACL reconstruction, although more targeted research is necessary.

We also found that a smooth function of patient age significantly predicted opioid administration and ROE. The likelihood of opioid administration rose as a function of age, leveling out around age 11. ROE seemed to level out later, increasing steadily until the late teens. The general age trends are not unexpected - opioids are administered more often and at higher doses in older children and adolescents [18,19].

Our findings identified that patients undergoing surgical treatment in an inpatient or observation unit were less likely to be administered opioids compared to those in an ambulatory setting. The differences in likelihood of opioid utilization may indicate different perioperative pain management protocols being used in these facilities. In a healthy patient, pain control may be prioritized over opioid stewardship, and alternative regimens may less commonly be implemented. Nevertheless, our findings suggest re-examination of opioid utilization practices in ambulatory ACL surgery may be warranted.

We estimated that patients with commercial insurance were not more likely to be administered opioids, but when administered, they had a greater ROE than patients without commercial insurance. We believe this difference could arise either directly or indirectly. On one hand, this dosing disparity could be a result of sub-optimal pain control for patients with a noncommercial payer or over-exuberant pain treatment for those with commercial insurance [20,21]. On the other hand, this disparity could be downstream of other effects of insurance status. Prior work has suggested that patients with commercial insurance may reach ACL reconstruction surgery more expediently than patients without commercial insurance [22], and patients with acute ACL injuries receive more opioids immediately after surgery compared to those with chronic ACL injuries [23]. Thus, differences in ROE could be due to delayed reconstruction in those with noncommercial insurance. Regardless of its source, this result underscores the need for further research in socioeconomic disparities in ACL treatment and rehabilitation.

Findings from this study indicate that perioperative administration of opioids in pediatric ACL reconstruction surgery is common at the typical hospital, but that non-narcotic perioperative pain management does occur in some settings. Neither administration rates nor ROE stably decreased for the typical hospital over the study period, contrary to our hypothesis. Hospital-level time trends demonstrated huge variability, both in level and trend (Figure 3).

There are several limitations to the internal validity of this study. First, the use of multi-modal pain regimens for surgically managed patients, notably regional anesthesia, was not assessed in this study. The effects we found may be partly or entirely mediated by the administration of non-opioid pain management at certain centers. Second, as an administrative data study, some measurement error in both patient characteristics and outcomes is expected. If this error was systematic, our patient and hospital-level findings may be compromised. However, the regularly conducted PHIS data quality checks greatly diminish the likelihood of such a threat. Third, the generalizability of this study may also be limited. The PHIS database represents only patients from tertiary care pediatric hospitals, which may not be representative of all pediatric patients undergoing operative treatment for ACL injuries. Morphine equivalent dose, although useful, remains a controversial construct due to the qualitative differences in opioid pharmacokinetics, pharmacodynamics, and tolerance [24].

## Conclusions

This ten-year administrative database study examining nationwide perioperative opioid administration to a pediatric orthopedic cohort found that patient gender, age, surgical setting, and hospital were predictive of opioid administration. Use and ROE during this period showed no indication of decline despite the sociopolitical climate currently surrounding opiates. The results of our study introduce novel findings that, similar to other pediatric fields and settings, variation in opioid exposure also exists during the perioperative period. Further efforts need to be taken to identify the rationale for this variability in patients undergoing similar surgical procedures.
